# The Limits of Inductive Reasoning for Clinical Evidence Appraisal: A Simulation Study

**DOI:** 10.7759/cureus.77047

**Published:** 2025-01-06

**Authors:** Steffen Mickenautsch, Veerasamy Yengopal

**Affiliations:** 1 Faculty of Dentistry, University of the Western Cape, Cape Town, ZAF; 2 Community Dentistry, University of the Witwatersrand, Johannesburg, ZAF

**Keywords:** clinical trial, error, inductive reasoning, simulation study, trial appraisal

## Abstract

Background

To establish the possible likelihood of a body of evidence, inductively judged to be of ‘low bias risk’/‘high-quality’ according to a limited set of appraisal criteria, of actually being error-free.

Methods

A total of 45 simulation trials were generated and randomly assigned to 0-5 errors out of a total of 65 error domains. The trials were then appraised for errors with a simulated appraisal tool consisting of five pre-specified error domains. Trial appraisal yielded either true positive, true negative, false negative or false positive results. From these values, the negative likelihood ratio (-LR) with 95% confidence interval (CI) was computed. -LR computation was repeated 25 times, each with newly generated random values for all 45 trials. The individual results of all 25 runs were statistically pooled. The pooled -LR result with 95% CI was interpreted as how likely a ‘low bias risk’/‘high-quality’ rated body of evidence is actually error-free.

Results

The pooled -LR was 0.84 (95% CI: 0.80 - 0.88, I^2^ = 0.0%). The result suggests that error-free evidence is only 1.2 times more likely to be rated as ‘low bias risk’/‘high-quality’ than evidence containing some form of error.

Conclusions

The likelihood of a ‘low bias risk’/‘high-quality’ rated body of evidence being actually error-free is small and the inductive generalization from any limited, pre-specified set of appraisal criteria rarely justifies a high level of confidence that a ‘low bias risk’/‘high-quality’ rating of clinical evidence reflects the true effect of a certain treatment without being affected by error.

## Introduction

Inductive reasoning is the process of generalizing from limited observations or facts [1]. It occurs during clinical evidence appraisal when a body of evidence (e.g. clinical trial(s), systematic review(s) of trials) is judged as being of ‘low bias risk’/‘high-quality’ because some of its characteristics are found to be in compliance with all criteria/items of a particular applied appraisal tool. Currently, common evidence appraisal tools [2-6] have adopted such inductive reasoning into their rationale.

Such reasoning has been criticized as being unjustifiable, because it is based on the unprovable assumption that since all the appraised evidence characteristics have been judged to be error-free, all characteristics of that evidence that were not appraised are not affected by error, too [7]. This assumption is sometimes justified by another limited set of observation, i.e. observations that verify such assumption, but which in turn are based on a similar assumption that any latter observations would not contradict the former, thus eventually leading to an infinite regress of invalid reasoning [8].

A ‘low bias risk’/‘high-quality’ rating is meant to express a high level of confidence that clinical evidence reflects the true effect of a certain treatment without being affected by error. However, error may be considered broadly as any mistaken conclusion or unintended outcome on material, observational, conceptual or discursive level [9] of which, for example, systematic error (bias) may be only a small subset of the observational error-type. Therefore, it is reasonable to assume that a very large number of error types may exist that cannot be appraised within any limited set of appraisal criteria of any appraisal tool. For example, the Catalogue of Bias Collaboration has so far listed 65 different bias (systematic error) domains that may affect clinical evidence [10]. In addition, it has been suggested that one single systematic error may already be sufficient to invalidate a body of clinical evidence [11].

For that reason, the aim of this simulation study was to establish the possible likelihood of a body of evidence, judged to be of ‘low bias risk’/‘high-quality’ according to a limited set of appraisal criteria, of actually being error-free.

This manuscript has been made available online as preprint in Research Square [12].

## Materials and methods

**Figure 1:**
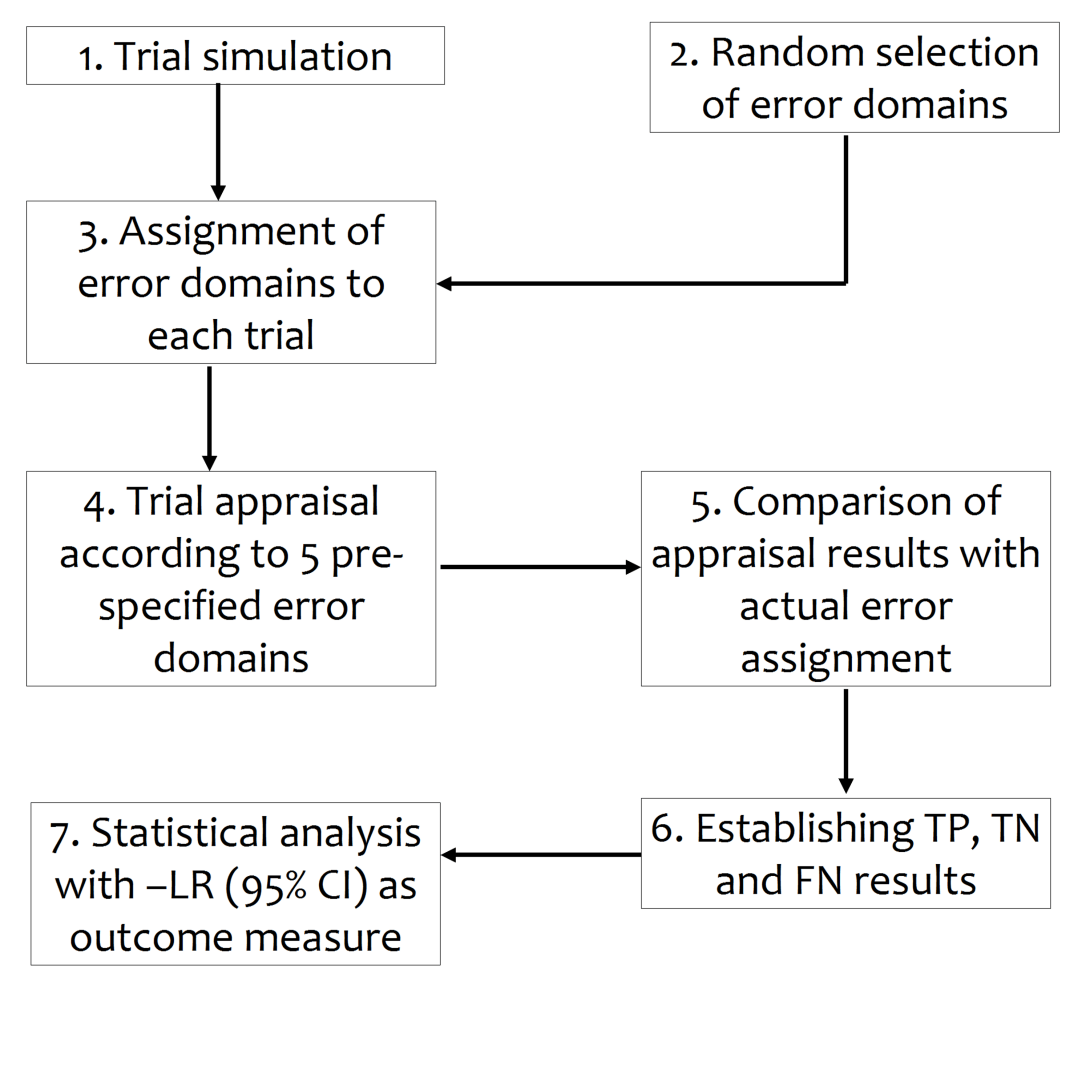
Flowchart of study method Trial appraisal result: TP = True positive, TN = True negative, FN = False negative; - LR = Negative likelihood ratio; CI = Confidence interval.

The protocol of our study was made available online prior to its start [12]. A flowchart of the followed study method is presented in Figure 1.

Our study was based on the premise that one single error (ɛ) can invalidate evidence [11] and that the number of all possible errors is not infinite (nɛ ≠ ∞). Based on these premises, the following assumptions were made:

The total number of all possible error domains is 65 (ɛ01 - ɛ 65) (Assumption 1). One body of evidence (further referred to as simulation trial) can be randomly affected by errors from 0-5 different domains (Assumption 2). Appraisal of the simulation trials can only be undertaken for the following five error domains that are randomly selected from that of the total set of 65 errors = ɛ03; ɛ013; ɛ018; ɛ23; ɛ46. Similar to Cochrane’s Risk of Bias tool / Version 2 (RoB 2) for five particular bias domains [2], these domains (according to Assumption 2) comprise a pre-specified tool, according to which all simulation trials in this study were appraised (Assumption 3). Trial appraisal will either yield a perfectly positive (trial with error) or negative outcome (trial without error) when one or more errors of the five pre-specified domains (ɛ03; ɛ013; ɛ018; ɛ23; ɛ46) were present in a particular simulation trial or not (Assumption 4). The five pre-specified error domains (ɛ03; ɛ013; ɛ018; ɛ23; ɛ46) included in the simulated appraisal tool are a random sample out of the set of all possible error domains (ɛ01 - ɛ 65) (Assumption 5). The distribution of errors from the set of all possible error domains (ɛ01 - ɛ 65) in the simulation trials is random (Assumption 6). Each error domain corresponds to only one single error (Assumption 7).

Since perfect accuracy of error identification in our simulation is assumed (see Assumption 4), the question of sample size-dependent precision did not arise. Hence, the number of simulation trials was set at 45, which - albeit arbitrary - was deemed sufficient for the purpose of this study. Within the context of this study, a simulation trial constituted a numbered row in an MS Excel file (see Appendices) to which errors from a number of error domains were assigned.

The number of 0, 1, 2, 3, 4 or 5 errors that can affect a trial (Assumption 3) was randomly assigned for each simulation trial. According to the number of assigned possible errors, a sample of error domains was randomly selected per trial from the total set (ɛ01 - ɛ 65), for example, Simulation trial 01/ randomly assigned number of errors = 3/ randomly selected error from the domains = ɛ01; ɛ024; ɛ39.

To assure that all sampling in this study was operator-independent, all random selection was undertaken by use of an online random number generator [13]. The generator’s simplistic version was used to generate the number of errors (0-5) per simulation trial, as well as for the random selection of the five pre-specified error domains for trial appraisal. The comprehensive version of the generator was used for randomly selecting the different error domains out of the total set of domains (ɛ01 - ɛ 65) with the following settings: Lower limit = 1; Upper limit = 65; Allow duplication of results? = No; Sort the results? = Ascend; Type of result to generate = Integer.

Trial appraisal yielded either true positive (TP), true negative (TN) or false negative (FN) results:

TP = Trial includes one or more errors from the domains included in the appraisal tool: ɛ03; ɛ013; ɛ018; ɛ23; ɛ46; TN = Trial does not include any error at all; FN = Trial includes one or more errors but not those from the domains included into the appraisal tool.

Trials with either TP or FN ratings were considered to be ‘low bias risk’/‘high-quality’ rated trials. The occurrence of a false positive (FP) appraisal result was not assumed and thus its value was always set at zero. In practice, any FP rating may be possible when trials are rated as biased on the basis of non-compliance with an appraisal criterion, yet the criterion itself is either not supported by empirical evidence of bias or the trial has been falsely deemed positive, due to some form of appraisal error. In line with Assumption 4, such a scenario was excluded in this simulation study.

From the TP, TN, FN and FP values of the 45 trials, the negative likelihood ratio (-LR) with 95% CI was computed as primary outcome measure of this study. In line with convention [14], the -LR in this study was adopted as the ratio of the probability of a trial affected by error (ɛ-trial) to be rated as a ‘low bias risk’/‘high-quality’ body of evidence divided by the probability of a trial not affected by error (non-ɛ-trial) to be rated as a ‘low bias risk’/‘high-quality’ body of evidence. (A ɛ-trial was considered a simulation trial to which errors from one or more error domains have been assigned. A non-ɛ-trial was considered a simulation trial with no errors.)

We were not able to compute a positive likelihood ratio (+LR), which is calculated by division of the FP value, because the occurrence of an FP appraisal result is not assumed in our simulation (i.e. set at zero) and thus would result in the mathematically impossible division by zero.

In order to reduce measurement error and in keeping with previous simulation scenarios [15], -LR computations were repeated 25 times (further referred to as “runs”), each with newly generated random values for all 45 trials. The individual -LR values of all 25 runs were statistically pooled by use of a random-effects meta-analysis based on the standard DerSimonian Laird random-effects model [16]. Between-run variance was computed using I^2^ - statistics and a 0.5-value was added to cells with zero values during computation.

The pooled -LR result with 95% CI was interpreted as how likely a ‘low bias risk’/‘high-quality’ rated body of evidence is actually error-free: -LR < 0.1 = highly, 0.1 - 0.2 = moderate, 0.2 - 0.5 = small but sometimes valuable, 0.5 - 1.0 = small and rarely valuable [17]. A negative likelihood ratio close to the value 1.0 indicated that a ɛ-trial is almost as likely to be rated a ‘low bias risk’/‘high-quality’ body of evidence than a non-ɛ-trial.

From the results, it was ascertained whether inductive generalization from limited appraisal criteria justifies a high level of confidence that a ‘low bias risk’/‘high-quality’ rating of clinical evidence reflects the true effect of a certain treatment without being affected by error.

## Results

The randomly generated number of errors and type of error domains per simulation trial for all 25 runs are listed in the Appendices (Sheet 1 Section 1) and the subsequent TP, TN, FN and FP values generated from the simulated trial appraisal per run are presented in Table 1.

**Table 1:** Trial appraisal results generated from the simulated trial appraisal per run TP = True positive; FP = False positive; FN = False negative; TN = True negative. RUN = Number of repetition.

Run	TP	FP	FN	TN
01	8	0	27	10
02	6	0	33	6
03	8	0	27	10
04	6	0	32	7
05	4	0	34	7
06	5	0	32	8
07	9	0	26	10
08	10	0	27	8
09	8	0	31	6
10	6	0	30	9
11	6	0	29	10
12	8	0	31	6
13	13	0	23	9
14	8	0	29	8
15	12	0	28	5
16	10	0	30	5
17	10	0	30	5
18	6	0	33	6
19	7	0	28	10
20	7	0	31	7
21	9	0	26	10
22	4	0	31	10
23	5	0	31	9
24	8	0	31	6
25	10	0	26	9

**Figure 2:**
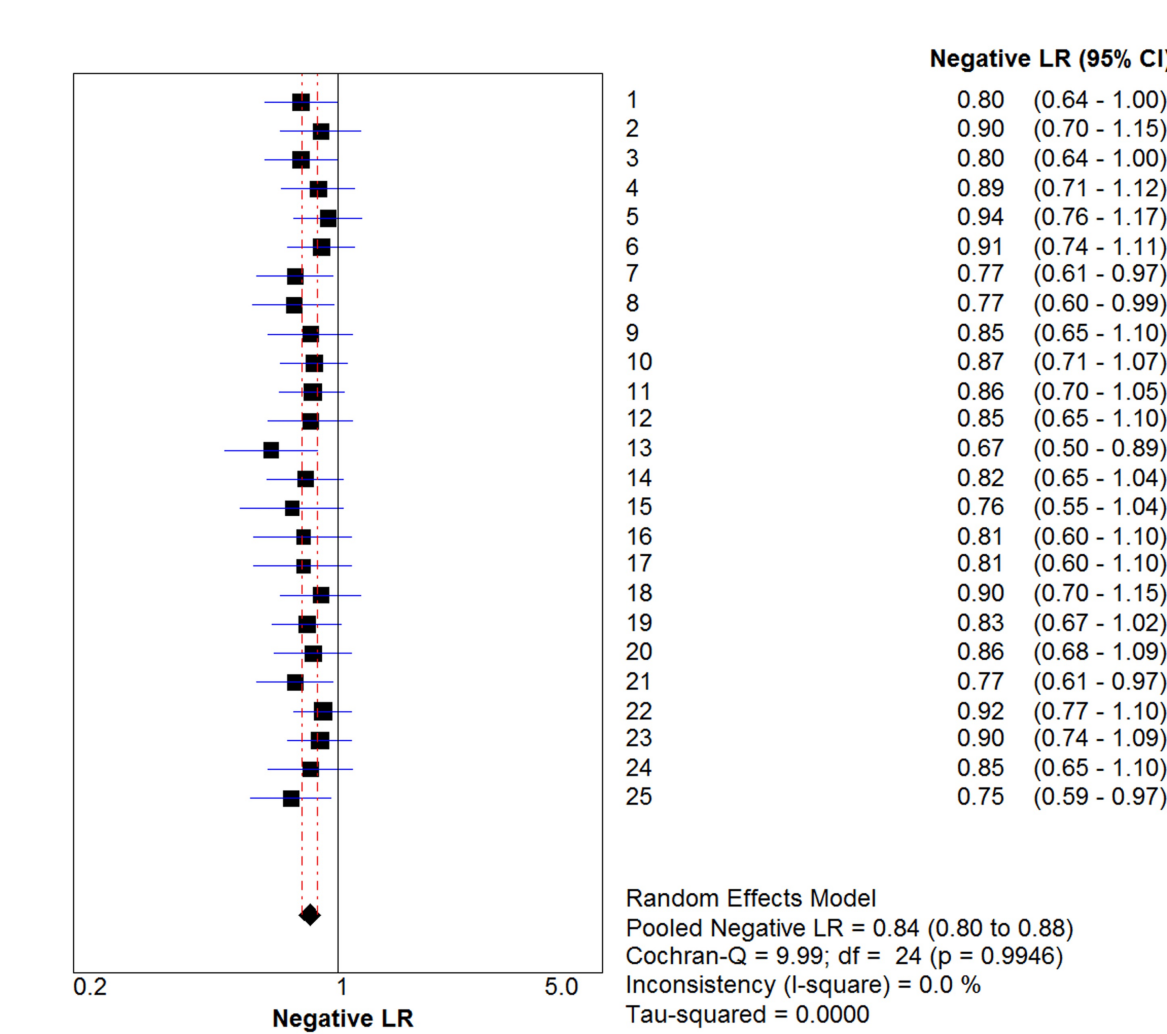
Negative likelihood ratios (LR) with 95% Confidence intervals (CI) per run

Figure 2 shows the computed negative likelihood ratios (-LR) with 95% CI per run. The pooled -LR was 0.84 (95% CI: 0.80-0.88, I^2^ = 0.0%).

The results of the step-wise meta-analysis of the 25 runs (Appendices, Sheet 2 Section 2) indicated that a higher number of runs would not have yielded a significantly different point estimate but would only have resulted in a narrower CI between the 0.80 and 0.89 values. Thus, the established pooled -LR was deemed sufficiently precise for the purpose of this simulation study.

The pooled negative likelihood ratio of 0.84 (95% CI: 0.80 - 0.88) suggests that non-ɛ-trials are only 1.2 times more likely to be rated as ‘low bias risk’/‘high-quality’ evidence than ɛ-trials. Hence, the likelihood of a ‘low bias risk’/‘high-quality’ rated body of evidence being actually error-free was found to be small and the inductive generalization from the limited, pre-specified set of appraisal criteria rarely justified a high level of confidence that a ‘low bias risk’/‘high-quality’ rating of clinical evidence reflects the true effect of a certain treatment without being affected by error.

## Discussion

The result of this simulation study suggests that the likelihood of a body of evidence, judged to be of ‘low bias risk’/‘high-quality’ according to a limited set of appraisal criteria, of actually being error-free is small. However, this result is largely dependent on the study’s premises and assumption that were set prior.

Limitation of study design

The study’s first premise that one single systematic error alone may suffice to invalidate a body of evidence was suggested in 2009 by Berger and Alperson based on theoretical considerations [11]. It is supported by empirical meta-epidemiological study results, which show that, although not all observed differences in clinical trial characteristics are associated with significant changes in effect estimates, some single differences, such as the lack of allocation concealment, appear indeed sufficient [18]. The latter may thus be considered as a single systematic error from a certain error domain (selection bias), sufficient to invalidate evidence, while differences in clinical trials that are not associated with significant changes in effect estimates may not be considered errors at all.

Our study’s second premise that the number of errors is finite seems more likely. However, in truth, we cannot really know, since it seems impossible to ever establish its actual number. To limit its number to 65 (Assumption 1) appears an underestimation, since it includes only the domains of systematic errors that are currently known [10], which in turn seems to be only a subset within the possible taxonomy of all (including unknown and unknowable) errors [9]. Accordingly, the number of errors that can affect a body of evidence may, although not infinite, be much larger. Consequently, the larger the total number of errors, the larger (and closer to the value 1.00) would our negative likelihood ratio have become and the less the actual distinction between a body of evidence, judged to be of ‘low bias risk’/‘high-quality’ from a ɛ-trial. Hence, the negative outcome of our study may still be regarded as a rather optimistic overestimation.

Our Assumption 2 limits the number of errors that can affect one body of evidence at the same time, to five. This may also be considered a generous underestimation and would have affected the result of our study in the same way as our second premise and Assumption 1. Assumption 3 reduces the number of pre-specified error domains in an appraisal tool to five. It must be considered that the more error domains were included in our simulated appraisal tool, the more errors would have been identified and resulted in larger TP values. This in turn would have decreased our negative likelihood ratio. However, limiting the number of error domains to five is in keeping with the number of systematic (bias) domains currently included in Cochrane’s RoB 2 tool [2], although some other tools, like AMSTAR 2 and GRADE, include a few domains more (Table 2).

**Table 2:** Examples of evidence appraisal tools that generalize from a limited set of applied criteria AMSTAR = A Measurement Tool to Assess systematic Reviews; GRADE = Grading of Recommendations, Assessment, Development, and Evaluations; RoB = Cochrane’s Risk of Bias tool; ROBINS-I = Risk Of Bias In Non-randomised Studies - of Interventions; ROBIS = Risk of Bias in Systematic Reviews

Trial appraisal tool	Appraisal of	Number of error domains	Overall judgment when all criteria are met (“verbatim”)
AMSTAR 2	Systematic reviews (Quality)	7	“Rating overall confidence in the results of the review / High / No or one non-critical weakness: the systematic review provides an accurate and comprehensive summary of the results of the available studies that address the question of interest” [5]
GRADE	Body of evidence (Quality)	5 (negative) + 3 (positive)	Quality of Evidence: High, if down rating according to all 5 (negative) domains plus up rating according to all 3 (positive) domains [6]
RoB 2	Clinical trials (randomized)	5	“Overall bias: Risk of bias judgment (low/high/some concerns) / “Low risk of bias - The study is judged to be at low risk of bias for all domains for this result” [2]”
ROBINS-I	Clinical trials (non-randomized)	3	“Judgment: Low risk of bias / The study is judged to be at low risk of bias for all domains” [4]
ROBIS	Systematic reviews (Bias risk)	4	“If the answers to all signaling questions for a domain are ‘‘yes’’ or ‘‘probably yes,’’ then level of concern can be judged as low … depending on the rating of the other signaling questions, the review may still be rated as ‘‘low risk of bias.” [3]

Assumption 4 assumes a perfect appraisal result within the limits of the applied appraisal tool. In practice, that may seldom be the case and this would introduce a further potential for errors, which would have negatively affected the pooled -LR value. We adopted only Assumptions 5 and 6 for random distribution, in order to assure operator independency in our study. The actual distribution of error may or may not follow a systematic format (where errors from different domains systematically cluster and interact with each other).

In summary, our prior set premises and assumptions rendered our simulation study an unrealistic optimistic overestimation (instead of a pessimistic underestimation) of reality. However, even despite such assumed unrealistic optimism, its result remains a negation of the validity of inductively generalizing from a limited set of appraisal criteria that a body of evidence is in truth error-free.

Discussion of study result

The outcome of our study provides some form of empirical evidence against the use of inductive reasoning in general and against its application during clinical evidence appraisal in particular. It appears to support the logical stance that inference from a limited number of observations, no matter how numerous, to universal (generalized) statements cannot be justified, because any conclusions drawn in this way may always turn out false [19].

The result of this study does seem to provide, albeit weak, support in defence of inductive reasoning that even though it is not possible to know with certainty whether a universal statement is true (e.g. that a body of evidence is completely error-free), it is at least possible to assign a higher probability for it when a sufficient number of supporting observations are made (e.g. when all applied appraisal criteria are complied with) [8]. The result in support of this proposition is weak because the pooled negative likelihood ratio of 0.84 (95% CI: 0.80 - 0.88) suggests that non-ɛ-trials are only 1.2 times more likely to be rated as ‘low bias risk’/‘high-quality’ evidence than ɛ-trials. In addition, such weak support would even disappear entirely if in reality the total number of possible errors would exceed 65 by far.

In addition, empirical evidence suggests a possibly even higher likelihood for error in ‘low bias risk’ rated clinical trials [20]. A total of 1070 published randomised control trials (RCTs) with median publication year: 2018; Interquartile range: 2013 - 2020, were statistically tested for selection bias using the “simulated comparator trial” (SCT) adjusted version of the I^2^ test [21]. The results showed a 6% higher likelihood for high selection bias risk in all RCTs that were previously rated as of “low bias” risk with Cochrane’s Risk of Bias, Version 2 (RoB 2) tool for bias domain 1: “Bias arising from the randomisation process” (-LR 1.06; 95%CI: 0.98 - 1.15) [20].

From a logical point of view, the proposition that inductive reasoning is at least supported by a higher probability of truth has been dismissed on the basis that it falsely applies the concept of event probability (for example, the probability to throw a ‘4’ with a six-sided dice is 1/6) to the concept of whether universal statements are true or false. Such application is erroneous because any assumption of any degree of validity between ‘true’ or ‘false’ would be in violation of the classical logical law of the excluded middle, which states that a statement can only be ‘true’ or ‘false’ without any option possible in-between [8].

However, even without considering its lack of logical soundness, any truth probability would technically require a ratio of the number of supporting observations to a reference sequence of the total number of observation not yet made. Such reference sequence appears unknowable or is at least difficult to determine [19] and, as our study shows, seems vulnerable to gross underestimation. This is further relevant in regard to the possible objection that errors should not be considered equal in their relevance and likelihood for clinical trials. Such differentiation of error types is of course possible, but only for known errors and only on the basis of a limited set of past observations. Both carry again the shortcomings of induction that lead to an infinite regress of reasoning. When unknown or even perhaps unknowable are considered together with known error types, no differentiation in error relevance and likelihood is possible.

Deductive reasoning as alternative for clinical evidence appraisal

The result of this study suggests that inductive reasoning during clinical evidence appraisal may lead to cases where erroneous bodies of evidence are rated as ‘low bias risk’/‘high-quality’. In contrast, deductive reasoning is the process of inferring from a universal statement (e.g. a body of evidence is corroborated, if it complies with all appraisal criteria) to single cases (e.g. a body of evidence does not comply with one criterion, therefore it is not corroborated) [1]. Such reasoning can provide a better alternative during evidence appraisal. As soon as one single error is identified, it can with a high level of confidence be inferred that clinical evidence is of ‘high bias risk’/’low-quality’ and thus cannot reflect the true treatment effect [7]. Evidence that has complied with all applied appraisal criteria to date is deductively considered as ‘corroborated’. Corroboration does not imply ‘low bias risk’/‘high-quality’ nor can corroborated evidence be considered more robust than evidence that has been found erroneous (i.e. falsified) because its full compliance with a particular set of appraisal criteria can give no certainty for compliance with any additional criterion in future. Corroboration indicates only that, so far, no error has been identified and therefore no reason has yet been established that would justify the rejection of that evidence for clinical guidance [7].

Against this background, ‘corroborated’ and ‘low bias risk’/‘high-quality’ evidence appears to be of equally importance for clinical guidance. Nonetheless, the result of our simulation study suggests that the difference between the two is not just semantic: A high likelihood has been demonstrated that, according to inductive reasoning, ‘low bias risk’/‘high-quality’ rated evidence can be equally affected by some form of error as ‘high bias risk’/’low-quality’ rated evidence.

## Conclusions

The likelihood of a ‘low bias risk’/‘high-quality’ rated body of evidence being actually error-free is small and the inductive generalization from any limited, pre-specified set of appraisal criteria rarely justifies a high level of confidence that a ‘low bias risk’/‘high-quality’ rating of clinical evidence reflects the true effect of a certain treatment without being affected by error. In order to reduce the risk of rating erroneous bodies of evidence during clinical evidence appraisal as ‘low bias risk’/‘high-quality’ based on inductive reasoning, a deductive reasoning approach is suggested, instead.

## Appendices

All data are fully available without restriction via:

https://data.mendeley.com/datasets/kxddgdtxnw/1
